# Computational Methods to Study DNA:DNA:RNA Triplex Formation by lncRNAs

**DOI:** 10.3390/ncrna9010010

**Published:** 2023-01-21

**Authors:** Timothy Warwick, Ralf P. Brandes, Matthias S. Leisegang

**Affiliations:** 1 Institute for Cardiovascular Physiology, Goethe University, 60590 Frankfurt, Germany; 2 German Centre of Cardiovascular Research (DZHK), Partner Site RheinMain, 60590 Frankfurt, Germany

**Keywords:** triplex, DNA–RNA triplex, DNA:DNA:RNA triplex formation, long non-coding RNA, interaction of DNA and RNA

## Abstract

Long non-coding RNAs (lncRNAs) impact cell function via numerous mechanisms. In the nucleus, interactions between lncRNAs and DNA and the consequent formation of non-canonical nucleic acid structures seems to be particularly relevant. Along with interactions between single-stranded RNA (ssRNA) and single-stranded DNA (ssDNA), such as R-loops, ssRNA can also interact with double-stranded DNA (dsDNA) to form DNA:DNA:RNA triplexes. A major challenge in the study of DNA:DNA:RNA triplexes is the identification of the precise RNA component interacting with specific regions of the dsDNA. As this is a crucial step towards understanding lncRNA function, there exist several computational methods designed to predict these sequences. This review summarises the recent progress in the prediction of triplex formation and highlights important DNA:DNA:RNA triplexes. In particular, different prediction tools (*Triplexator*, *LongTarget*, *TRIPLEXES*, *Triplex Domain Finder*, *TriplexFFP*, *TriplexAligner* and *Fasim-LongTarget*) will be discussed and their use exemplified by selected lncRNAs, whose DNA:DNA:RNA triplex forming potential was validated experimentally. Collectively, these tools revealed that DNA:DNA:RNA triplexes are likely to be numerous and make important contributions to gene expression regulation.

## 1. Introduction

A huge portion of the human transcriptome is not translated into proteins, and instead persists as so-called non-coding RNAs. The most common class of non-coding RNA is long non-coding RNA (lncRNA). LncRNAs are defined as RNAs longer than 200 nucleotides without apparent potential to code for proteins. They share mRNA-like features but are often less well conserved and expressed compared to protein-coding transcripts. Interestingly, the majority of lncRNAs appear to be expressed in a cell- or tissue-specific manner [1]. Despite the fact that most lncRNAs remain uncharacterised, numerous examples of physiologically important lncRNA have meanwhile been reported [2]. Functionally, lncRNAs are involved in different cellular processes. These include transcriptional regulation, post-transcriptional regulation (e.g., splicing), structural organisation and genome integrity. Of particular interest is that lncRNAs usually exert their functions through interactions with proteins, small molecules, metabolites, other RNAs or even DNA [1,2].

**Figure 1:**
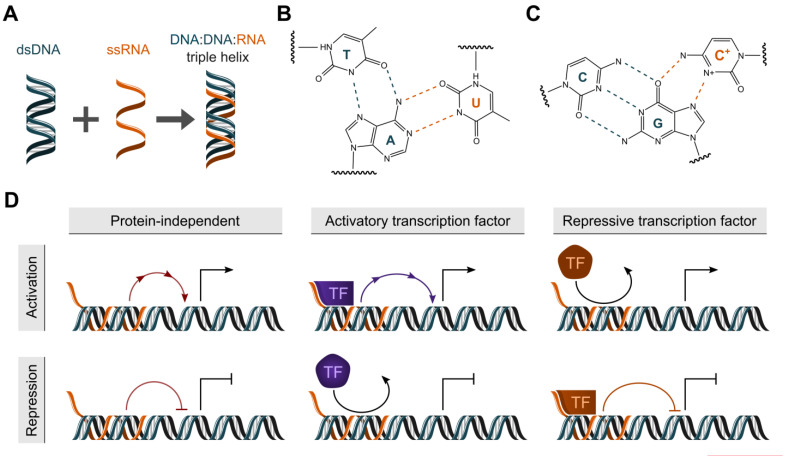
Overview of DNA:DNA:RNA triple helix formation (**A**) Schematic of DNA:DNA:RNA triple helix formation between double-stranded DNA and single-stranded RNA. (**B**,**C**) Canonical Watson–Crick and Hoogsteen (red) base pairings which permit the formation of DNA:DNA:RNA triple helices. (**D**) Putative mechanisms by which DNA:DNA:RNA triple helix formation permits the control of gene expression via interactions with gene loci and transcription factors.

Interactions between an RNA and DNA can occur in either heteroduplex (DNA:RNA) or triplex (DNA:DNA:RNA) conformations (Figure 1A) [1]. Where R-Loops are considered heteroduplexes formed by the displacement of a DNA strand by an RNA [3], triplexes are formed by the accommodation of single-stranded RNA (ssRNA) in the major groove of double-stranded DNA (dsDNA) [4]. In the context of a triplex, the ssRNA is referred to as a triplex-forming oligonucleotide (TFO), whereas the major groove of the target dsDNA is named the triplex target site (TTS) [5]. It is methodologically challenging to distinguish between R-loops and triplexes. One experimental approach is an enzymatic digestion of the nucleic acids with RNase H, which preferentially digests the RNA strand within R-loops but not in triplexes [6,7]. Within triplexes, the binding of the nucleic acids occurs via Hoogsteen or reverse Hoogsteen hydrogen bonds (Figure 1B,C), which are weaker than Watson–Crick bonds, but provide more flexibility. Depending on the nucleotide composition of the RNA strand, a purine-rich sequence of DNA is bound by the RNA strand in a parallel or antiparallel manner [8]. A variety of biophysical and biochemical methods can be applied to study triplexes, including circular dichroism- (CD) and nuclear magnetic resonance-spectroscopy (NMR) [9,10,11,12] and electrophoretic mobility shift assays (EMSA) [13]. To identify triplexes in a cellular context, triplex-seq or triplex capture assays have been developed [13,14]. So far, several lncRNAs have been reported to participate in DNA:DNA:RNA triplex formation. Among these are *FENDRR* [15], *KHPS1* [16], *MEG3* [9], *PAPAS* [17], *SARRAH* [11] and *HIF1α-AS1* [12]. However, the number of studies investigating triplex formation in living cells and therefore in a physiologically or disease-relevant context is still limited. Although methods such as Chromatin-Associated RNA sequencing (ChAR-seq) [18], global RNA interactions with DNA by deep sequencing (GRID-seq) [19], in situ mapping of RNA–genome interactome (iMARGI) [20], RNA ends on DNA capture (Red-C) [21], RedChIP [22], RNA And DNA Interacting Complexes Ligated and sequenced (RADICL-Seq) [23] and triplex-seq [13] have been developed, the accurate genome-wide analysis of DNA:DNA:RNA triplex formation is still a domain of computational prediction.

Prediction of DNA:DNA:RNA triplex formation is a key step in driving mechanistic hypotheses of lncRNA function (Figure 1D). Establishment of accurate predictive tools has been a challenge in the field for the past decade. This is predominantly due to the relative scarcity of appropriate data to train complex predictive models. A number of algorithms have been proposed which instead implement DNA:DNA:RNA base pairings which have been reported from in vitro experiments. Whilst the reasoning for this implementation is clear, benchmarking studies have shown the performance of these tools in the accurate recall of RNA–DNA interactions to be modest [24]. It is perhaps unsurprising that in vitro DNA:DNA:RNA base pairings are limited in their prediction of RNA–DNA interactions taking place in a cellular context with high accuracy. Numerous confounding factors are present in the cell, and the process surrounding triplex formation is complex and dynamic. Chromatin conformation and secondary RNA structures are likely to play key roles in where and how triplexes form. Recently, next-generation sequencing data detecting triplex-forming RNA and DNA have become available [13]. This information could therefore serve as an input to machine learning models and captures triplex formation in a cellular context. The outputs of these models have subsequently been implemented in the prediction of both triplex-forming sequences and genome-wide RNA–DNA interactions. Notably, despite divergent approaches, each proposed tool has been used successfully to support lncRNA research.

## 2. Computational Methods to Identify Triplex-Forming lncRNAs and their DNA Target Sites

### 2.1. Triplexator: The First Triplex Prediction Tool

**Figure 2:**
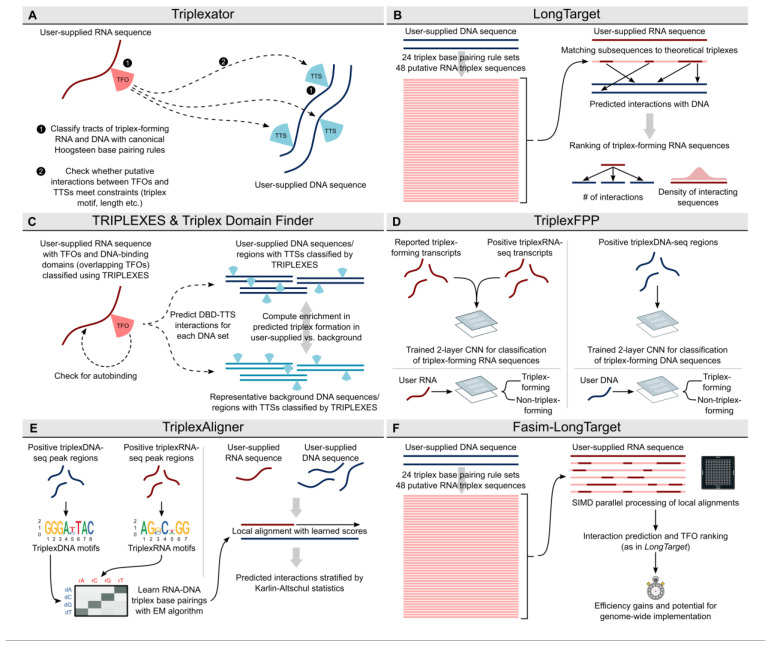
Computational tools used to predict the formation of DNA:DNA:RNA triple helices (**A**) Schematic representing the implementation of *Triplexator*. *Triplexator* classifies putative triplex-forming sequences in RNA (triplex-forming oligonucleotides, TFOs) and DNA (triplex target sites, TTSs) sequences prior to predicting potential interactions. **(B)** Implementation of *LongTarget. LongTarget* implements observed in vitro DNA:DNA:RNA base triplets to generate candidate triplex-forming RNA sequences. Local alignment is used to classify subsequences of user-input RNA as triplex-forming regions. (**C**) Workflow of *TRIPLEXES* and *Triplex Domain Finder. TRIPLEXES* classifies potential triplex-forming subsequences of input DNA and RNA (DNA-binding domains, DBDs). *Triplex Domain Finder* detects the statistical enrichment of predicted triplex formation between an input RNA and set of DNA regions versus appropriate background regions. (**D**) Training of the convolutional neural networks (CNNs) used in *TriplexFPP.* Known triplex-forming RNAs along with enriched RNA and DNA regions of triplex-sequencing data are used to train classifiers of triplex-forming RNA or DNA. (**E**) The development of *TriplexAligner. TriplexAligner* implements probabilistic RNA–DNA base pairings learned by expectation–maximisation (EM) from enriched triplexRNA and triplexDNA motifs detected in triplex-sequencing data as scoring matrices in local alignment between RNA and DNA sequences. Results are stratified using Karlin–Altschul statistics. (**F**) The workflow of *Fasim-LongTarget*, which enhances the computational performance of *LongTarget* through the use of SIMD (single instruction, multiple data) parallel processing of local alignments.

Multiple methods have been developed so far to predict the triplex-forming potential of RNAs (Figure 2A–F). *Triplexator* represents the first published piece of software for the prediction of DNA:DNA:RNA triplex formation [25] (Figure 2A). The method focuses on identifying regions of DNA and RNA which are likely to participate in triplex formation based on canonical DNA:DNA:RNA binding rules [26]. Given an input set of ssRNA and dsDNA, *Triplexator* defines candidate TFOs and TTSs, which satisfy a set of constraints including—but not limited to—length, non-canonical triad occurrence and guanine occurrence in the potential triplex. These constraints are relative to the set of triplex motifs, namely the pyrimidine motif, purine motif and purine–pyrimidine motif. By utilising these discrete motifs as templates for triplex formation, candidate TFOs and TTSs may be easily identified from user-supplied sequences. Following this, *Triplexator* identifies putative triplex interactions between the classified TFOs and TTSs by detecting maximal tracts of subsequences, which follow any one of the aforementioned triplex motifs. Tracts are considered to end when the triplex-forming rule sets are no longer followed, or the constraints used in the classification of TFOs and TTSs are breached. Using this approach, *Triplexator* returns maximally scoring predicted triplex interactions between maximally scoring TFOs and TTSs.

### 2.2. KHPS1 as an Example for Triplex-Mediated Transcriptional Activation and the Interchangeability of Triplex-Forming Regions

**Figure 3:**
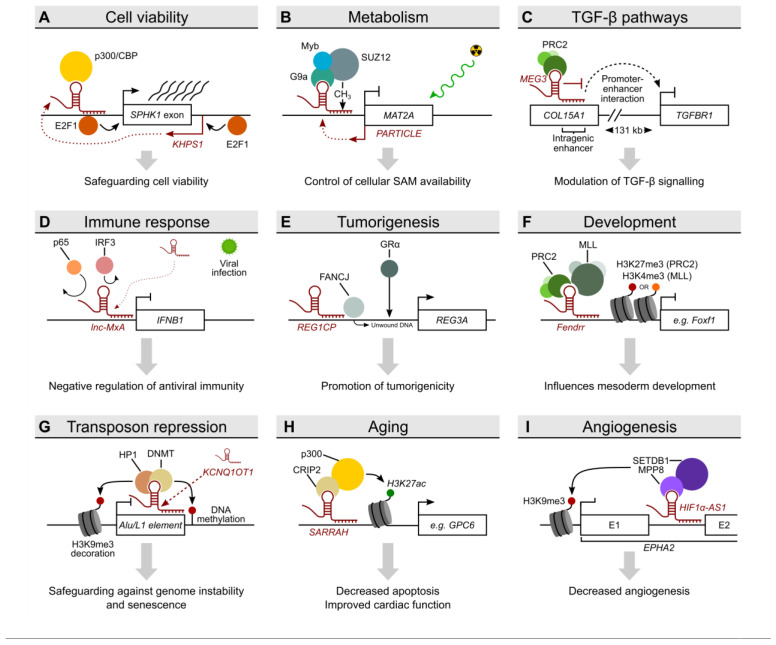
DNA:DNA:RNA triplex interactions and their proposed mechanisms of action (**A**) Triplex formation in *cis* by the lncRNA *KHPS1.* Through interactions with the transcription factors p300/CBP and E2F1, *KHPS1* enhances the transcription *SPHK1*. (**B**) Binding of the antisense lncRNA PARTICLE at the promoter of its sense gene. The transcription of the gene *MAT2A* is downregulated by *PARTICLE*-associated transcription factors. (**C**) Triplex formation by *MEG3* at a distal enhancer of the gene *TGFBR1*. This leads to the downregulation of gene expression, potentially via the actions of the polycomb repressive complex 2 (PRC2) complex which interacts with the *MEG3* transcript. (**D**) Formation of a DNA:DNA:RNA triple helix at the promoter of *IFNB1* by the lncRNA *lnc-MxA* upon viral infection. This results in the interruption of the binding of the transcription factors p65 and LSD1 to the region, thereby preventing upregulation of the target gene. (**E**) Association between *REG1CP* and the DNA helicase FANCJ at a distal promoter site of *REG3B*. This interaction permits DNA unwinding and gene upregulation by the glucocorticoid receptor. (**F**) Triplex formation by lncRNA *Fendrr*. Through interactions with the chromatin modifiers PRC2 and MLL, *Fendrr* facilitates the decoration of target genes such as *Foxf1* with repressive marks H3K27me3 and H3K4me3. This leads to the repression of *Fendrr* target genes. (**G**) Binding of the lncRNA *KCNQ1OT1* in conjunction with its protein interaction partners HP1 and DNMT leads to genome-wide repression of transposable element transcription via H3K9me3 decoration and DNA methylation. (**H**) The lncRNA *SARRAH* forms DNA:DNA:RNA triple helices at multiple loci. It activates transcription via recruitment of the transcription factors p300 and CRIP2, which deposit H3K27ac at target loci. (**I**) DNA:DNA:RNA triplex formation by the lncRNA HIF1α-AS1 at specific gene loci represses target gene transcription by the recruitment of members of the HUSH complex. This leads to the formation of repressive chromatin at target loci and results in the downregulation of target genes such as *EPHA2* and *ADM*.

The lncRNA *KHPS1* activates gene transcription in *cis* through triplex formation [16] (Figure 3A). *KHPS1*, which is transcribed antisense to *SPHK1*, binds to the *SPHK1* promoter—as shown by a pull-down assay, triplex capture assays and EMSA—and recruits the histone acetyltransferase p300/CBP. Acetylation of the histones around the *SPHK1* promoter increases promoter accessibility, leading to the transcriptional activation of *SPHK1* through the transcription factor E2F1. Thereby, triplex formation by *KHPS1* promotes cell proliferation, in turn inhibiting E2F1-induced apoptosis. In a follow-up study [27], the authors provided evidence that the triplex-mediated anchoring of *KHPS1* leads to the transcription of an eRNA (*eSPHK1*), which is required for the transcription of *SPHK1*. Furthermore, the authors suggested that the concept of triplex formation relies on the interchangeability of triplex forming sequences and triplexes as molecular anchors. This concept was exemplified by replacement of the TFO of *KHPS1* by the TFO of *MEG3*, which consequently links *KHPS1* to the *MEG3* target gene *TGFBR1* instead of *SPHK1*.

### 2.3. PARTICLE Links Triplex Formation to Irradiation, a Model System for Cellular Stress

O’Leary et al. identified the lncRNA *PARTICLE* (promoter of MAT2A-antisense radiation-induced circulating ncRNA), which is transcribed antisense to the promoter of Methionine adenosyltransferase 2A (*MAT2A*) and which is induced by low doses of ionising radiation. With the help of *Triplexator*, the authors predicted that *PARTICLE* forms a triplex with the *MAT2A* gene promoter, which was experimentally validated by surface plasmon resonance (SPR) experiments. Conceptually, *PARTICLE* represses *MAT2A* promoter activity and *MAT2A* expression through recruitment of PRC2, which methylates CpG islands within the triplex-forming site [28] (Figure 3B). In a follow-up study [29], the authors performed another in silico analysis of *PARTICLE* triplex target sites, this time using the mouse and human genomes and the *Triplex Domain Finder* tool. Using SPR and EMSA in conjunction with RNase H treatment, O’Leary et al. provided evidence that *PARTICLE* forms a triplex with another target, the tryptophan domain containing oxidoreductase *WWOX*. After irradiation, *PARTICLE* was predominantly localised in the nucleus and associated with chromosome 16, where the *WWOX* gene is located. As in the case for *MAT2A*, *PARTICLE* negatively regulates *WWOX* gene expression as demonstrated by knockdown and over-expression experiments, suggesting that *PARTICLE* potentially recruits additional epigenetic complexes as in the case of *MAT2A*.

### 2.4. MEG3-Dependent Triplex Formation Is Involved in the Regulation of Signalling Pathways

Another example of a lncRNA involved in triplex formation is Maternally Expressed 3 (*MEG3*) [9]. Mondal et al. identified repressive chromatin-associated lncRNAs by using a modified chromatin RIP followed by high-throughput sequencing protocol (ChRIP-Seq) and analysed how *MEG3* recognises its target genes. An EZH2–*MEG3* interaction is important to repress the expression of several genes involved in the transforming growth factor β pathway (Figure 3C). In that particular study, motif enrichment analysis as well as *Triplexator* identified GA-rich sequences present in *MEG3* target loci and *MEG3* RNA as critical sequences. EMSA with and without RNase A/H digestion, CD spectroscopy, triplex capture assays and triplex ChIP-qPCR validated triplex formation of *MEG3* at its target genes *TGFBR1*, *TGFBR2* and *SMAD2*. Using different *MEG3* mutants, the authors showed that the interactions of *MEG3* with chromatin and PRC2 are mediated by distinct sequences within the lncRNA. This study demonstrated that triplex formation by lncRNAs is able to regulate gene expression in *trans*.

### 2.5. Lnc-MxA and REG1CP: Triplex Formation Meets Immune Response and Cancer

Li et al. reported on an lncRNA, *Lnc-MxA,* capable of triplex formation on a gene involved in immune responses [30]. Using *Triplexator*, *Lnc-MxA* was predicted to bind to the promoter of *IFNB1* (Figure 3D), which was validated by Chromatin Isolation by RNA Purification (ChIRP)-qPCR, in vitro triplex pulldown assay, EMSA with RNase treatment and different mutants. The authors found that during viral infection, *lnc-MxA* inhibits interferon β transcription by blocking the binding of the transcription factor p65 and IRF3 to the *IFNB1* promoter.

Another example of the use of *Triplexator* is lncRNA *REG1CP*. This lncRNA links triplex formation to tumorigenesis through an enhancer complex involving the FANCJ helicase (Figure 3E). *REG1CP* forms a triplex with the *REG3A* gene as validated by EMSA and RNase A/H treatment, in vitro binding assays and different triplex mutants [31]. Upregulation of *REG1CP* occurs in colorectal cancer cells and is associated with poor outcomes.

### 2.6. LongTarget

The next publication of a computational tool for the prediction of triplex formation was that of *LongTarget* [32] (Figure 2B). Instead of following only canonical Hoogsteen base pairing rules [33], *LongTarget* implements an extended set of reported DNA:DNA:RNA base pairing rules with the intent of capturing potential triplex interactions which may go undetected by *Triplexator*. Base pairings for which biophysical evidence had been reported, as well as those for which a triplex has been observed [34], were incorporated into *LongTarget*. The result of this approach was 24 triplex rule sets, of which 6 reflected Hoogsteen base pairing rules and the remaining 18 covered potential reverse Hoogsteen binding.

The algorithm itself works by constructing ideal triplex RNA sequences against the DNA (one per DNA strand) per triplex base-pairing rule, per user-supplied genomic region of interest. This results in 48 potential RNA sequences which would be assumed to form DNA:DNA:RNA triplexes with the dsDNA supplied to the algorithm. In order to determine whether the candidate RNA sequence supplied by the user is likely to interact with the genomic region in-question, the sequence is compared against the ideal candidate sequences using local alignment [35]. The configuration of the local aligner to report multiple alignments means that multiple putative TFOs may be reported along the length of the user-supplied RNA sequence. TFOs are subsequently ranked by several metrics. One of these is the number of DNA:DNA:RNA triplex interactions which each TFO is predicted to participate in, with TFOs involved in more interactions deemed to be more convincing. Another metric used to rank TFOs is how densely triplex-forming sequences are distributed across the breadth of the TFO. Dense, recurrent triplex-forming sequences are considered to be more convincing candidates.

Triplexes predicted by *LongTarget* are also annotated with in vitro DNA:DNA:RNA base triplet stability values. These values have been reported per base triplet [34,36], and *LongTarget* reports a summed value for the breadth of the predicted triplex. To assign a specificity value to the best-ranked TFO and its predicted interactions, triplex prediction is also carried out using shuffled versions of the user-supplied sequences.

Several studies have exemplified the use of *LongTarget* as a promising triplex prediction tool. Zhao et al. showed that LncRNA *DLX6-AS1* binds to the promoter of *DLX6* forming triplexes [37], and Ou et al. demonstrated that lncRNA *CDKN2B-AS1* forms triplexes at the promoter of *CDKN2B* [38]. Both validated their findings with triplex pulldown/capture assays, EMSA with RNase treatment and mutated TFOs. Interestingly, *FENDRR* (FOXF1 Adjacent Non-Coding Developmental Regulatory RNA) was one of the first lncRNAs shown to form triplexes and to recruit PRC2 to control developmental processes [15] (Figure 3F). Although this finding by Grote et al. was achieved without using *LongTarget*, the tool was used later in another study showing that *FENDRR* additionally forms triplexes with the *DRP1* promoter in hypoxic pulmonary artery endothelial cells [39]. In that study, the triplex forming sequences predicted by *LongTarget* were experimentally verified by ChIRP-qPCR, EMSA and RNase A/H treatments. The data suggested that *FENDRR* repressed *DRP1* expression through triplex formation, thereby increasing *DRP1* promoter methylation.

### 2.7. TRIPLEXES

As interest in DNA:DNA:RNA triplex formation continued to grow, along with the advent of novel wet-lab methods for detection of RNA–DNA interactions, the demand for the accurate prediction of triplex formation grew in turn. Similarly to *Triplexator* [25], *TRIPLEXES* [5] works by initially establishing putative TFOs and TTSs in user-supplied RNA and DNA sequences, respectively (Figure 2C). The classification of these regions is performed against a similar background of constraints to *Triplexator*. Namely: error rate relative to a triplex motif, consecutive errors, occurrence of guanine residues and length of the region. A key difference between *TRIPLEXES* and *Triplexator* is that the TFOs assigned by *TRIPLEXES* are initially limited to seeds of a defined length, controlled by a parameter. These seed regions are subsequently matched to the DNA sequence according to the same triplex motifs used by *Triplexator*, resulting in a set of seed triplexes. These are then extended using a heuristic algorithm, and any overlapping interactions are merged. *TRIPLEXES* also searches for regions of potential auto-binding of supplied RNA-sequences, which reflect cis interactions between a transcript and its gene locus. Such interactions have been linked to the regulation of gene expression, with examples including the lncRNAs *Fendrr* [15] and *Khps1* [16].

### 2.8. Triplex Domain Finder

*Triplex Domain Finder* is a framework for the statistical stratification of genomic regions and their propensity for forming triplexes with a user-supplied RNA [5] (Figure 2C). The method works by comparing predicted triplex formation between given transcripts and candidate regions of the genome, compared to a background set of genomic regions. Rather than working at the level of individual TFOs (computed by either *Triplexator* or *TRIPLEXES*), *Triplex Domain Finder* merges overlapping TFOs to form what are termed as DNA-binding regions of transcripts. The total number of TTSs mediated by a single DNA-binding region may then be enumerated by summing the total triplex interactions of each TFO incorporated into the region. This summed total is then statistically compared to the number of triplex interactions predicted between the DNA-binding domain and a representative set of background genomic regions. Any statistically enriched DNA-binding domains are returned as putative triplex-forming domains of the transcript. *Triplex Domain Finder* was used as a triplex-prediction tool in two lncRNA studies. First, Kalwa et al. demonstrated with EMSA and RNase H treatment the predicted regions for *PCDH7* and *HOXB2* to form triplex structures with HOTAIR domain II [40], and second, Kuo et al. used TDF to detect known and novel DNA binding domains of *MEG3* and *FENDRR* and newly identified GATA6-AS to form triple helices and affecting cardiac mesoderm differentiation [5].

In practice, the combination of *Triplexator*/*TRIPLEXES* and *Triplex Domain Finder* may be used to study whether a set of user-supplied genomic regions (e.g., promoters, peaks) are likely to be triplex targets of a transcript-of-interest. *Triplex Domain Finder* returns both empirical and false discovery rate-adjusted [41] *p*-values to summarise this. Additionally, genomic regions and multiple input RNAs can be stratified by the number of interactions they are predicted to be involved in, normalised nominally by sequence lengths.

### 2.9. Triplex Domain Finder as a Tool to Analyse Large Datasets

In a global analysis to identify DNA-associated RNAs [13], *Triplex Domain Finder* was used to determine whether these RNAs have the potential to form triplexes. Sentürk-Cetin et al. reported a large set of RNAs, among them RNAs from non-coding and coding loci, to be associated with DNA. Interestingly, the results suggest that triplex formation could be a general mechanism of RNA-mediated target-site recognition. Triplex formation seems to preferentially occur at open and active chromatin regions (around promoters), and the majority of RNAs engage in *trans* interactions with DNA. Among other triplex-forming RNAs, validation experiments concentrated on the lncRNA *NEAT1*, which was associated with DNA sequences from *FLI1*, *GRIK4* and *CYP4F22* in EMSA experiments. Also here, the presence of complex formation after treatment with RNase H excluded the possibility that the retardation of electrophoretic mobility was due to heteroduplex formation.

In a second study, the lncRNA *KCNQ1OT1* was shown to be important for DNA methylation and H3K9me3 histone decoration on *KCNQ1OT1*-targeted transposons [42] (Figure 3G). *Triplex Domain Finder* was used to predict the triplex-forming potential of lncRNA *KCNQ1OT1*. The authors identified 12 triplex-forming domains within *KCNQ1OT1*, with 9 of these being L1 sequences and 7 found within a repeat-rich region of the lncRNA. *Triplex Domain Finder* predicted more than 1600 *KCNQ1OT1* ChIRP-Seq binding sites to be potential triplex target sites. Interestingly, the L1 RNA sequence derived from *KCNQ1OT1* was shown to form a triplex with L1 or Alu DNA. This interaction was abolished by the replacement of the poly(A) in L1 RNA with poly(G). Using a specific CRISPR knock-in inserting the responsible TFO into the non-repeat-rich region, the authors demonstrated that TFOs in *KCNQ1OT1* are sufficient to guide the lncRNA to its targets, thereby enabling their methylation.

### 2.10. SARRAH: Triplex Formation Associated with Aging and Myocardial Infarction

In the following example, triplex formation was studied in the context of aging. The conserved lncRNA *SARRAH* (SCOT1-antisense RNA regulated during aging in the heart), also known as *OXCT1-AS1*, was found to be downregulated in aged mice as well as infarcted hearts. The lncRNA was also shown to be important for the survival of cardiomyocytes [11]. Interestingly, overexpression of *SARRAH* in mice enhanced their recovery from acute myocardial infarction. The authors identified that *SARRAH* has triplex-forming potential using *Triplex Domain Finder*, and they validated triplex formation at the *GPC6* locus using NMR spectroscopy for both human and murine *SARRAH/Sarrah* transcripts (Figure 3H). Other triplex target genes were identified after the downregulation of *SARRAH*. As in the case of *KHPS1*, *SARRAH*-mediated triplexes probably serve to activate gene expression: induction of NRF2 and the binding of CRIP2 and p300 facilitated the transcriptional activation of *SARRAH* target genes.

### 2.11. HIF1α-AS1 Is a Triplex-Forming lncRNA Validating the Concept of Interchangeability

*HIF1α-AS1* is a lncRNA transcribed in antisense direction of the physiologically important transcription factor HIF1α, whose triplex-forming potential was recently reported by us [12]. A search for DNA-associated lncRNAs using the triplex-seq data set published by Sentürk-Cetin et al. [13] revealed that lncRNA *HIF1α-AS1* is a triplex- and DNA-associated lncRNA in endothelial cells [12]. *HIF1a-AS1* was important to limit endothelial spheroid outgrowth and to promote apoptosis, and its expression was decreased in specific pulmonary diseases and glioblastoma. We identified TFOs and TTSs of *HIF1α-AS1* using ATAC-seq and *Triplex Domain Finder*, and validated the triplex formation of *HIF1α-AS1* at the *EPHA2* and *ADM* gene loci using NMR spectroscopy, CD spectroscopy, EMSA, CRISPR and several RIP and ChIP experiments involving RNase A/H. Moreover, as in the case of *KHPS1*, we could show the interchangeability of triplex-forming sites by replacing the TFO of *HIF1α-AS1* with the TFO of *MEG3* and observing the subsequent interaction of *HIF1α-AS1* at the *MEG3* triplex target *TGFBR1*. Mechanistically, triplex formation of *HIF1α-AS1* leads to the transcriptional repression of *EPHA2* and *ADM* through the recruitment of the epigenetic HUSH complex members MPP8 and the histone methyltransferase SETDB1 (Figure 3I).

### 2.12. TriplexFFP

All of the tools described up to this point either implemented canonical Hoogsteen and reverse Hoogsteen DNA:DNA:RNA base pairing rules [5,25,26,33], or fixed triplex base combinations which have been observed under in vitro conditions [32,34]. However, with the development of next-generation sequencing methods designed to capture chromatin-associated RNAs or RNA-associated DNA [13], using machine learning algorithms to predict triplex formation became feasible.

*TriplexFPP* was the first method published with this aim [43]. By training a convolutional neural network (CNN) [44] using RNA or DNA sequences reported to be triplex-forming, extracted features can subsequently be used to predict any other sequence as triplex-forming (Figure 2D).

For the training of any machine learning model, the choice of input data is key. In case of *TriplexFFP*, the input data consisted of a mixture of published triplex-forming RNA sequences such as *MEG3* [9], PARTICLE [28], *MIR100HG [45]*, *FENDRR* [15] and *HOTAIR* [40], as well as *Triplexator*-identified TFOs present in triplexRNA-seq peak regions [13]. For negatively labelled data, lncRNAs which had *Triplex Domain Finder*-predicted DNA-binding domains but no experimentally validated triplex formation were used. To train the CNN for the prediction of triplex target sites in DNA sequences, RNA-associated genomic regions from Sentürk-Cetin et al. [13] were used as a positive dataset. A set of random promoter regions with an identical length distribution to the positive data was used as negatively labelled input data. *TriplexFPP* was effective at classifying the input data, although the performance in recall of further triplex-forming transcripts and genomic regions remained unexplored. *TriplexFPP* does not aim to predict RNA–DNA interaction pairs, although in practice, positively predicted regions could be used as input to any one of the other tools described herein.

### 2.13. TriplexAligner

We recently published *TriplexAligner*, a computational tool which also uses machine learning to predict the formation of DNA:DNA:RNA triplexes [46]. The approach to the problem in *TriplexAligner* is, however, fundamentally different to all other tools. Whereas all other tools use canonical DNA:DNA:RNA base pairing rules to some extent, *TriplexAligner* instead implements rules learned by expectation–maximisation from triplexRNA-sequencing and triplexDNA-sequencing data [13] (Figure 2E). These learned RNA:DNA base pairings—termed codes—are then implemented as substitution matrices in local alignment between user-supplied RNA and DNA sequences.

Where the input data to the machine learning model used in the development of *TriplexAligner* were similar to that of *TriplexFPP* (chromatin-associated RNA peaks or RNA-associated chromatin peaks from [13]), *TriplexAligner* did not filter and preselect regions using *Triplexator*. Instead, motif enrichment analysis [47] was utilised to identify short, repetitive sequence elements which were assumed to be constituent subsequences of triplex-forming regions. Following the characterisation and filtering of RNA and DNA triplex motif sets, these were used as input to an expectation–maximisation algorithm [48]. The function of this algorithm was to return probabilistic RNA–DNA pairing matrices, which support the pairing of a number of RNA–DNA motif pairs. The in vitro stability of the reported codes was assessed by annotation with DNA:DNA:RNA triplex base pair stabilities [49].

In order to use the learned RNA–DNA base pairing rules in the prediction of DNA:DNA:RNA triplex formation, the matrices were implemented in a local alignment program. User-supplied RNA and DNA sequences are aligned using each of the eight reported RNA–DNA base pairing matrices, with the output being alignments stratified by E-value [50].

Given the lack of explicitly canonical or in vitro DNA:DNA:RNA base pairing rules implemented in *TriplexAligner*, validation of the tools ability to accurately predict RNA-DNA interactions was carried out using genome-wide datasets arising from RADICL-sequencing [23] and RedC [21]. In these validations, *TriplexAligner* outperformed both *LongTarget* and *Triplex Domain Finder* in the accurate recall of RNA–DNA interactions. In addition, triplex formation experiments performed in the characterisation of *SARRAH* could be recapitulated using *TriplexAligner*. Finally, predicted triplex forming sequences were biophysically validated using CD- and NMR-spectroscopy.

### 2.14. Fasim-Longtarget

The prediction of genome-wide triplex formation remains an open challenge in the field. In an attempt to fill this gap, a modified version of *LongTarget* was developed which takes advantage of advances in local alignment algorithms [51]—*Fasim*-*LongTarget* [52]. Given that the implementation of *LongTarget* requires 48 separate local alignments per RNA–DNA pair, the computational gains to be made by implementation of modified local alignment algorithms is sizable. *Fasim*-*LongTarget* computes parallelised local alignments using the Single-Instruction Multiple-Data (SIMD) instruction [53], resulting in marked gains in computational efficiency whilst maintaining a similar prediction accuracy to *LongTarget* during validation with genomic binding sites of *MEG3* [9], *NEAT1* and *MALAT1* [54] (Figure 2F).

## 3. Conclusions and Outlook

We are currently beginning to understand the importance of triplex formation for cell function. The remarkable progress in the field during the last decades has provided evidence that lncRNAs form triplexes with important physiological consequences. As such, lncRNA-mediated triplex formation contributes to gene expression control, which could even be indirectly important for disease development. Due to the extensive number of lncRNAs in the genome and the possibility of hundreds and thousands of DNA interaction sites, there is no doubt that lncRNA-mediated triplex formation offers a great potential and perspective for future RNA-based therapeutics.

However, as is the case for lncRNAs, other RNAs can also form triplexes. Physical evidence for triplex formation has been provided for miRNAs, potentially explaining why miRNAs could also lead to an upregulation of target genes. Paugh et al. claimed that miRNAs thereby regulate gene expression since higher expression of triplex-forming miRNAs was more frequently associated with increased gene expression [55]. Triplex formation can also function as a cis-acting regulatory mechanism at the human β-globin and FAU locus involving triplex-forming RNAs (potentially evolving from protein-coding transcripts) from the same locus [7]. Cis-regulatory roles of triplex forming RNAs were also observed for promoter-associated RNAs. The interfering RNA from the minor promoter of the dihydrofolate reducates gene *DHFR* forms a triplex at the *DHFR* gene’s major promoter region, disturbing transcription factor binding, which leads to decreased *DHFR* expression [56]. Another example is the inhibitory effect of pRNA, which is complementary to the rDNA promoter, on rDNA expression through triplex-mediated recruitment of DNA methyltransferase DNMT3b, DNA methylation and interference of the target site of the transcription factor TTF-I [57]. This underlines the fundamental roles of DNA:DNA:RNA triplex formation and highlights their potential druggability in many disease scenarios.

Since only a relatively small fraction of lncRNAs have been investigated so far, triplex formation of the majority of lncRNAs remains unstudied. Current research is just beginning to reveal the great importance of lncRNA triplex formation in gene expression control. Whether triplexes contribute to other processes beyond gene regulation, such as the regulation of alternative splicing, the recruitment of accessory factors, the mediation of DNA (or RNA) repair events, genome instability and senescence or the assembly of complexes of higher ordered structures remains to be determined. Experimentally validated triplex forming lncRNAs are highly important for testing predictive methods. Such lncRNAs used for this purpose include *MEG3, Fendrr, HOTAIR, SARRAH, NEAT1* and *MALAT1*. It should be noted that using these lncRNAs imposes a positive selection bias on any computational validation, though. Comparing different tools according to their predictions of interactions of single lncRNAs is also very narrow, when all-to-all data arising from assays such as RADICL-seq and RedC are available.

As an increasing number of lncRNAs and their potential triplex formation are investigated, the importance of choosing appropriate computational tools becomes more important. In this review, we have highlighted the concepts and methodologies underlying each method. With the exception of *TriplexFPP*—which only classifies RNA and DNA as triplex forming or not—all the tools described herein report predicted interactions between user-supplied RNA and DNA. The regions of lncRNAs which are predicted to undergo triplex formation are highly important, because they allow a targeted validation of predicted interactions by wet-lab methods. In *Triplexator* and *Triplex Domain Finder*, triplex-forming regions of RNA are predicted prior to the interaction prediction according to potential Hoogsteen base pairing motifs. In *LongTarget* and *TriplexAligner*, these regions can be extracted following the prediction of interactions based on the total number of interactions that the region is predicted to be involved in. A large-scale comparison between triplex-forming regions of RNAs is yet to be undertaken in a benchmark study at the time of this review.

When examining benchmark studies on this topic, it is clear that the use of purely canonical DNA:DNA:RNA base pairing rules for the prediction of triplex formation is insufficient [24,46]. *TriplexAligner*, which follows a divergent approach and uses rules learned from triplexes present in a cellular context, outperforms both *Triplex Domain Finder* and *LongTarget* in recall of genome-wide RNA-DNA interactions [46]. The benchmarking during the development of *TriplexAligner* used both RADICL-seq and RedC RNA–DNA interactions. This revealed that *TriplexAligner* was more accurate than the other tools across both interaction sets. *Triplexator*/*Triplex Domain Finder* outperformed *LongTarget* in recall of RADICL-seq interactions, but not RedC interactions. This follows the benchmark results reported by Antonov et al. (2019), where *Triplexator* marginally outperformed *LongTarget* in the prediction of *MEG3*–DNA interactions as detected by ChOP-seq. However, in the manuscript describing *Fasim-LongTarget*, both *LongTarget* and *Fasim-LongTarget* were reported to outperform *Triplex Domain Finder* in recall of the RNA–DNA interactions of *MEG3*, *NEAT1* and *MALAT1*. The use of different datasets and parameters across these experiments make reporting a definitive gold-standard tool difficult. Future benchmarking of these tools should focus on global RNA–DNA interaction data—available from RADICL-seq and RedC—where *TriplexAligner* outperformed the other tested programs.

This shows that the complexities of triplex formation in vivo are not completely reflected in canonical triplex base pairings. Notably, the rulesets learned in the course of the development of *TriplexAligner* also include a mixture of canonical and non-canonical DNA:DNA:RNA base pairings, each with a foundation in *in vivo* triplex formation [13]. Currently missing from *TriplexAligner*, though, is a statistical framework akin to that implemented by *Triplex Domain Finder*. Therefore, these two tools seem to be the most promising for the identification of accurate, statistically ranked triplex predictions. Given that *TriplexAligner* was validated using global RNA–DNA interaction data, it seems best-suited to large-scale exploratory analyses of triplex formation. Computational runtime should also be considered as a factor when running these types of jobs, though. For smaller analyses on select RNAs and DNA regions, either or both of the tools could be used, given that both have been tested using experimentally validated triplex-forming lncRNAs. However, it should be noted that the outputs of the tools differ considerably, and direct comparison of results is not trivial. This may result in more confusion over results if tools are used together in a meta-analysis style.

Also of consideration should be the user-friendliness and usability of tools for prediction of triplex formation. In terms of the runtimes of the different prediction tools, a concrete comparison is currently missing from the literature. In the benchmark study by Antonov et al., *Triplexator* was reported to be faster to execute than *LongTarget* [24]. However, *Fasim-LongTarget* has subsequently been reported as having a shorter runtime than *Triplex Domain Finder* [52]. *TriplexAligner* is yet to be time profiled, and such benchmarks would require the standardization of data input, parameter selection and hardware. With the exception of *TriplexAligner*, all of the software described herein must be run from the command line, presenting an obstacle to use by non-experts. *TriplexAligner* is available as an *R* package, and as such is more accessible to users, with many non-bioinformaticians having some experience with *R*. To be truly accessible to users, the implementation of a triplex prediction tool as a web server would be optimal.

In the field of DNA:DNA:RNA triplex formation, the relationship between wet-lab research and computational prediction is extremely close. Where the prediction of triplex formation has been an enabling factor for mechanistic research into lncRNA functions, future development and improvement in predictions of genome-wide and context-dependent triplex formation is reliant on the continued development and establishment of biochemical methods for detecting these events. Whilst working from a basis of in vitro DNA:DNA:RNA base pairings, the future direction of predicting triplex formation is in the development of machine learning models which take into account the complexities and dynamics of living cells. In addition, usability issues of predictive computational methods hamper the development of the field. Among these are genome-wide scalability, user-friendliness and downstream interpretation of results. Better implementation of these aspects will result in more effective and more broadly used predictive tools which will in turn facilitate future lncRNA research.

## Data Availability

Not applicable.
